# Passively Q-Switched Er-Doped Fiber Laser Based on Bentonite Clay (Al_2_H_2_O_6_Si) Saturable Absorber

**DOI:** 10.3390/mi15020267

**Published:** 2024-02-13

**Authors:** Haroon Asghar, Umer Sayyab Khalid, Muhammad Sohail, Tahani A. Alrebdi, Zeshan A. Umar, A. M. Alshehri, Rizwan Ahmed, M. Aslam Baig

**Affiliations:** 1 National Centre for Physics, Quaid-i-Azam University Campus, Islamabad 45320, Pakistan; 2 International Collaborative Laboratory of 2D Materials for Optoelectronics Science and Technology of Ministry of Education, Institute of Microscale Optoelectronics, College of Electronics and Information Engineering, Shenzhen University, Shenzhen 518060, China; 3 Department of Physics, College of Science, Princess Nourah Bint Abdulrahman University, P.O. Box 84428, Riyadh 11671, Saudi Arabia; 4 Department of Physics, King Khalid University, P.O. Box 9004, Abha 61413, Saudi Arabia

**Keywords:** Q-switched operation, pulsed fiber lasers, erbium-doped fiber lasers, bentonite clay material, saturable absorber

## Abstract

This paper presents the investigations toward the direct use of bentonite clay (Al_2_H_2_O_6_Si) nanoparticles to act like a saturable absorber (SA) for the Q-switched pulse operation of an erbium-doped fiber laser (EDFL). The measured results reveal that with the incorporation of bentonite clay nanopowder as a SA, an EDFL is realized with a Q-switching mechanism starting at a pump power of 30.8 mW, and a Q-switched emission wavelength was noticed at 1562.94 nm at 142 mW pump power. With an increased pump from 30.8 mW to 278.96 mW, the temporal pulse parameters including minimum pulse duration and maximum pulse repetition rates were reported as 2.6 µs and 103.6 kHz, respectively. The highest peak power, signal-to-noise ratio, output power and pulse energy were noticed to be 16.56 mW, 51 dB, 4.6 mW, and 47 nJ, respectively, at a highest pump power of 278.96 mW. This study highlights the significance of bentonite clay (Al_2_H_2_O_6_Si) nanoparticles as a potential candidate for a saturable absorber for achieving nonlinear photonics applications.

## 1. Introduction

In recent years, fiber lasers have emerged as the most promising technologies for high-power and high-quality laser output sources [1]. Among various types of fiber lasers, continuous-wave (CW) erbium-doped fiber lasers (EDFLs) have drawn significant considerations due to their unique properties, including their wide gain bandwidth, efficient pumping, compact size, and low noise performance [2,3]. In addition, CW-EDFLs are attractive for their ability to amplify signals transmitted over long distances [4] and emit light at specific wavelengths for spectroscopy [5] and remote sensing [6]. The implementation of EDF as an active gain medium in a fiber laser makes it suitable to operate in the near-infrared region of spectra, offering its useful applications that require high-power, short-pulsed output at wavelengths of around 1550 nm, which is highly desirable in optical communications [7]. The EDFLs can be easily incorporated with other fiber-optic components, such as amplifiers and modulators, making them highly versatile tools for various applications. To generate ultrashort, high-intensity pulses with particular pulse duration, and repetition rates, the mechanisms of Q-switching [8], mode locking [9], and hybrid mode locking [10], are paramount.

In the following, we investigated the Q-switching pulse characteristics of EDFLs. Q-switched EDFLs have many applications in a variety of fields containing material processing [11], spectroscopy [12], medical [13], optical communication [14], range finding [15], and remote sensing [16]. Active Q-switching and passive Q-switching are two further techniques used in laser physics to generate short pulses of high-intensity light. The fundamental difference between these two techniques is the way in which the pulses are generated from the laser sources. Active Q-switching mechanism comprises the use of an external component, like an electro-optic modulator or an acoustic-optic modulator, to control the Q-factor of a laser cavity. By rapidly varying the Q-factor, the laser cavity can be made to fill up with photons, leading to a high-intensity pulse being emitted. Passive Q-switching involves the incorporation of a SA within the laser cavity. The SA acts as a gatekeeper, preventing the laser from emitting until the intensity reaches a certain threshold. Once the threshold is reached, the absorber quickly saturates, allowing a high-intensity pulse to be emitted. Passively Q-switched SAs have several advantages over other types of Q-switching mechanisms which include simple design, high stability, low cost, high pulse energy, wide wavelength range, and compatibility with fiber lasers. 

Several types of SAs are commonly used in fiber lasers, and a few of them are semiconductor saturable-absorber mirrors (SESAMs) [17], graphene [18], carbon nanotube [19], topological insulators [20], transition metal dichalcogenides [21], oxide-based thin-films [22,23], black-phosphorus [24] and quantum-dots [25]. Typically, these SAs materials lead toward higher absorption at lower optical intensities and become transparent at relatively higher intensities making these materials suitable for the generation of ultrashort pulses of light. Bentonite clay (BNC) is a type of absorbent aluminum phyllosilicate clay containing mostly montmorillonite. The nanoscale form of BNC has gained significant consideration due to its unique properties. BNC, with varying magnetic, optical, and electrical properties, is an ideal material for sensor development and image formation contras [26,27]. The molecular formula of BNC is Al_2_H_2_O_6_Si with a band gap of 2.26 eV [26]. BNC is also used as a coating in optical surfaces to act as a protective layer or anti-reflective coating. BNC also has the potential to use index-matching purposes in optical systems to adjust the medium refractive index relative to others. In addition, BNC has better thermal stability, which could be beneficial for the fabrication of high-threshold-based SAs. Therefore, due to these unique chemical, physical, and tunable optical properties, the implementation of BNC inside laser cavities to act as a SA needs to be explored in detail.

In this study, we demonstrate a facile method for the BNC nanoparticles deposition on the fiber facet, which serves as an SA in EDFLs. The deposition process involves immersing the fiber ferrule into a BNC nanoparticle powder, resulting in a successful coating of the ferrule. By incorporating the BNC-based SA into the EDFL system, we achieved a Q-switched mechanism at a deficient threshold power of 30.8 mW. Our findings also indicate that the system exhibited a maximum repetition rate of 103.6 kHz, a minimum pulse duration of 2.6 µs, an output power of 4.6 mW, a pulse energy of 47 nJ, and a peak power of 16.56 mW at a maximum power of 278.96 mW. These results exhibit the potential of BNC nanopowder as a promising material for developing efficient and cost-effective SAs in laser systems.

## 2. Deposition of Bentonite Clay Saturable Absorber (SA)

**Figure 1:**
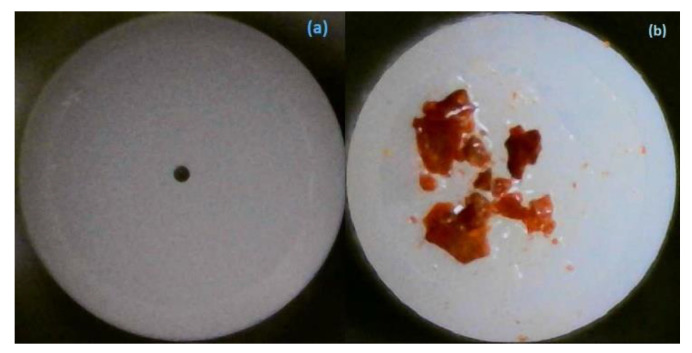
The fiber facet (**a**) without and (**b**) with deposition of BNC-SA.

BNC nanoparticles investigated as an SA in this study were purchased from Sigma Aldrich (Germany), Hydrophilic Bentonite (CAS-No: 1302-78-9), and were used as received. The BNC nanoparticles were first drawn on the filter paper and were then deposited on the core of the fiber by employing the adhesion effects of the index-matching gel. The index-matching gel offers the advantage of the ease of BNC nanopowder deposition on the fiber facet and has a similar refractive index (1.463) as that of single-mode fiber (SMF). The BNC-SA deposited on the fiber facet was then attached with other clean SMF via a FC/PC connector. A BNC-SA device was then ready to use, once incorporated in the laser cavity. The deposition was achieved at room temperature, and the absorber thickness was optimized. The optimization of SA was performed by changing the thickness of SA on the fiber facet until a better performance of EDFL was achieved. The deposition of BNC-SA at the surface of the fiber ferrule is shown in Figure 1a,b.

## 3. Synthesis and Characterization of Bentonite Clay Nanoparticles

**Figure 2:**
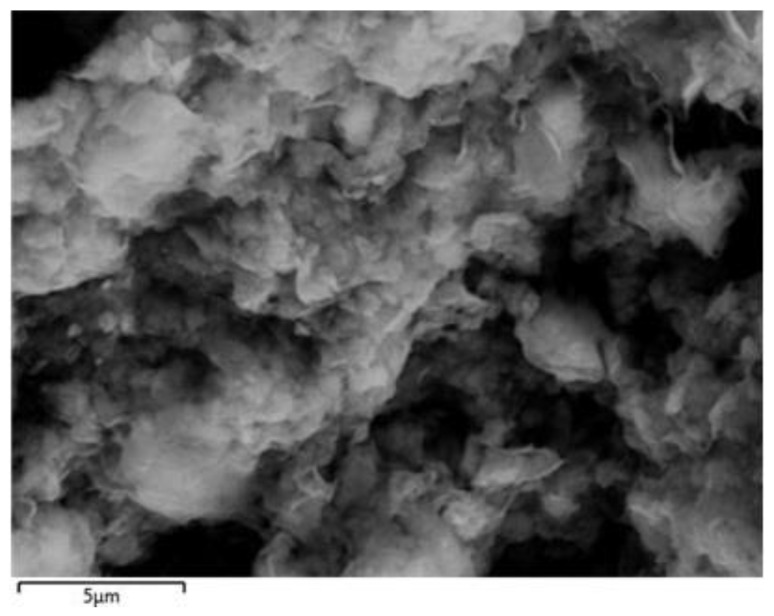
FESEM image of bentonite clay nanoparticles.

**Figure 3:**
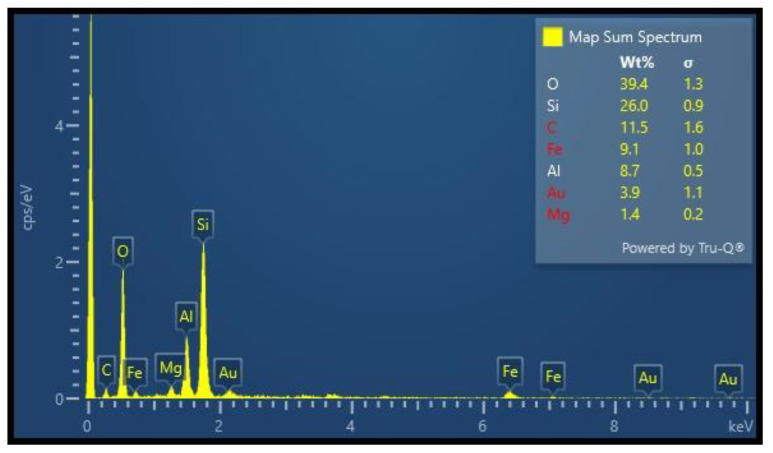
The energy-dispersive X-ray spectroscopy of bentonite clay nanoparticles.

Figure 2 and Figure 3 illustrate the comprehensive characterization of BNC nanoparticles including a field-emission scanning electron microscope (FESEM) and energy-dispersive X-ray spectroscopy (EDX) techniques. The morphology of the BNC sample was carried out using a FESEM. The micrograph of the BNC sample is presented at 5 µm resolution, which specifies the structure and morphology of BNC. The BNC is in a 2D layered structure, as shown in Figure 2. The agglomeration of BNC powder can be observed in Figure 2, but the average size of BNC sample lies in the nm scale.

The elemental concentration of the BNC sample was conducted via an EDX technique. An Oxford-X-MAX-N-20 EDX instrument in combination with a SEM functioning at 30 keV was employed to study the chemical composition of a sample under investigation. Figure 3 depicts the results of an EDX analysis on the BNC sample; peaks corresponding to Al (8.7%), O (39.4%), and Si (26.0%) were identified. The EDX instrument could not detect the hydrogen because of its low atomic number and weak X-ray emission. C (11.5%) was used as a substrate, and Au (3.9%) was used to prepare the sample during SEM analysis. Other elements Fe (9.1%), and Mg (1.4%) are impurities in the sample during the preparation process of the material.

## 4. Modulation Depth of Bentonite Clay Nanoparticles

**Figure 4:**
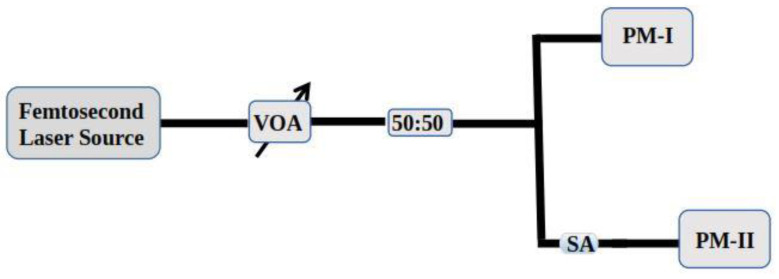
Experimental arrangements to measure nonlinear characteristics of BNC-SA; acronyms—VOA: variable optical attenuator; SA: saturable absorber; PM: power meter.

**Figure 5:**
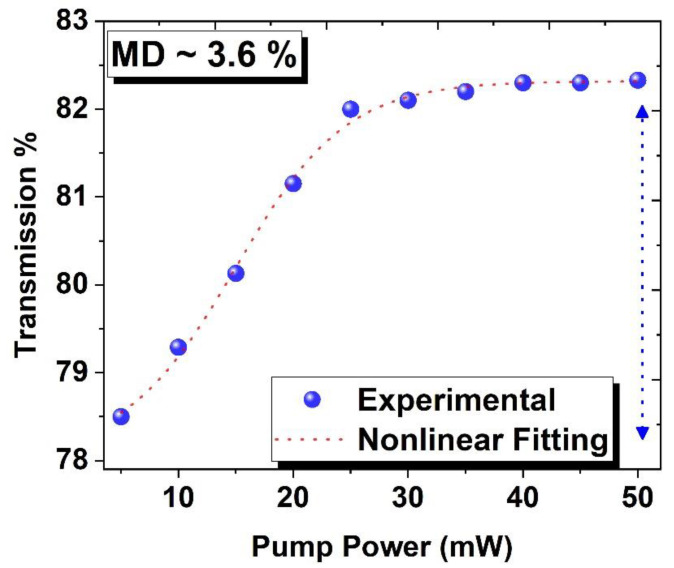
The nonlinear transmission curve of BNC-SA emitting at 1560 nm.

In the following section, the nonlinear-optical characteristics of the BNC-SA device have been examined via a balanced twin-detector technique. The schematics of the experimental arrangements are depicted in Figure 4. A stable femtosecond mode-locked laser source at 1561.2 nm center wavelength, operating at 14.5 MHz repetition rates, with a corresponding 912 fs pulse duration was used. The intensity of optical light was adjusted with the help of a VOA (variable optical attenuator), and then optical light was split equally via a 3 dB coupler. After that, two separate power meters were employed to investigate the power. The first power meter was employed to investigate the power directly for reference and the second power meter was employed to collect power when SA was adjusted inside the laser cavity. The experimental (solid blue spheres) and non-linear curve fitting (dashed red line) data are depicted in Figure 5.

The nonlinear saturable absorption characteristics are desired from the curve fitting of experimental data employing the following saturable absorption model [28]:

$$T(I)=1-\Delta T*\exp(-\frac{I}{I_{sat}})-T_{ns}$$

Here, *T*(*I*) represents the coefficient of intensity-dependent transmission, Δ*T* indicates the modulation depth, saturation intensity is denoted by *I_sat_*, and *T_ns_* is non-saturation loss. The nonlinear transmission curve yields a modulation depth of 3.6%. These measurements show that BNC-based SAs can be used for pulse generation.

## 5. Experimental Setup of Erbium-Doped Fiber Laser

**Figure 6:**
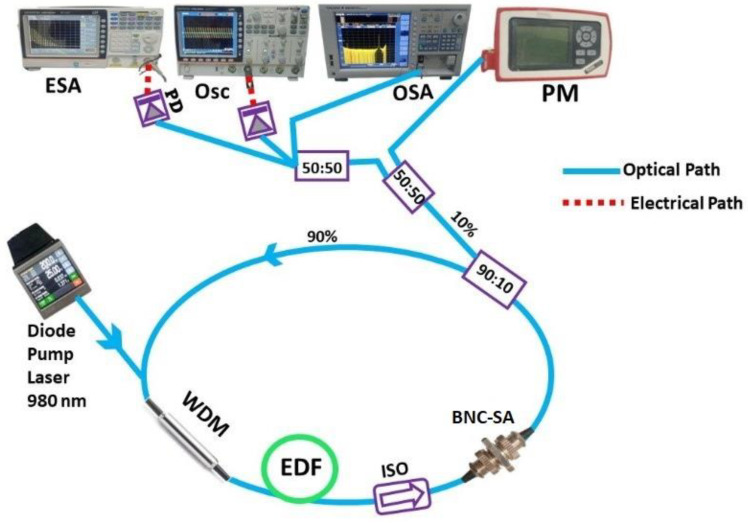
A schematic of the experimental setup of a Q-switched EDFL system subject to BNC-SA.

A schematic of the Q-switched EDFL system subject to BNC-SA is illustrated in Figure 6. The experiment involved a diode pump at a 980 nm wavelength to pump an EDF-based ring cavity using a 980/1550 nm WDM. The EDF length was fixed to 10 m, with a 6 dB/m of peak absorption at a 1530 nm wavelength with a 0.24 m numerical aperture. The overall cavity length was approximated around 12 m. The next end of the doped fiber was spliced with an isolator that was fixed in a ring cavity to keep oscillations of optical signal in one direction. The prepared BNC-SA was incorporated into the laser cavity after the isolator, and a 90/10 splitter was utilized for the division of optical light into two parts. For the analysis, the electric spectrum analyzer (GW-INSTEK, GSP-9330) attached with a 5 GHz bandwidth InGaAs-photodiode (DET08CFC-Thorlabs) was used for the measurement of RF spectra. Optical spectra and pulse train signatures were captured employing an optical spectrum analyzer (YOKOGAWA, AQ6370D) and a digital oscilloscope (GW INSTEK, GDS-3504), respectively.

## 6. Results and Discussion

**Figure 7:**
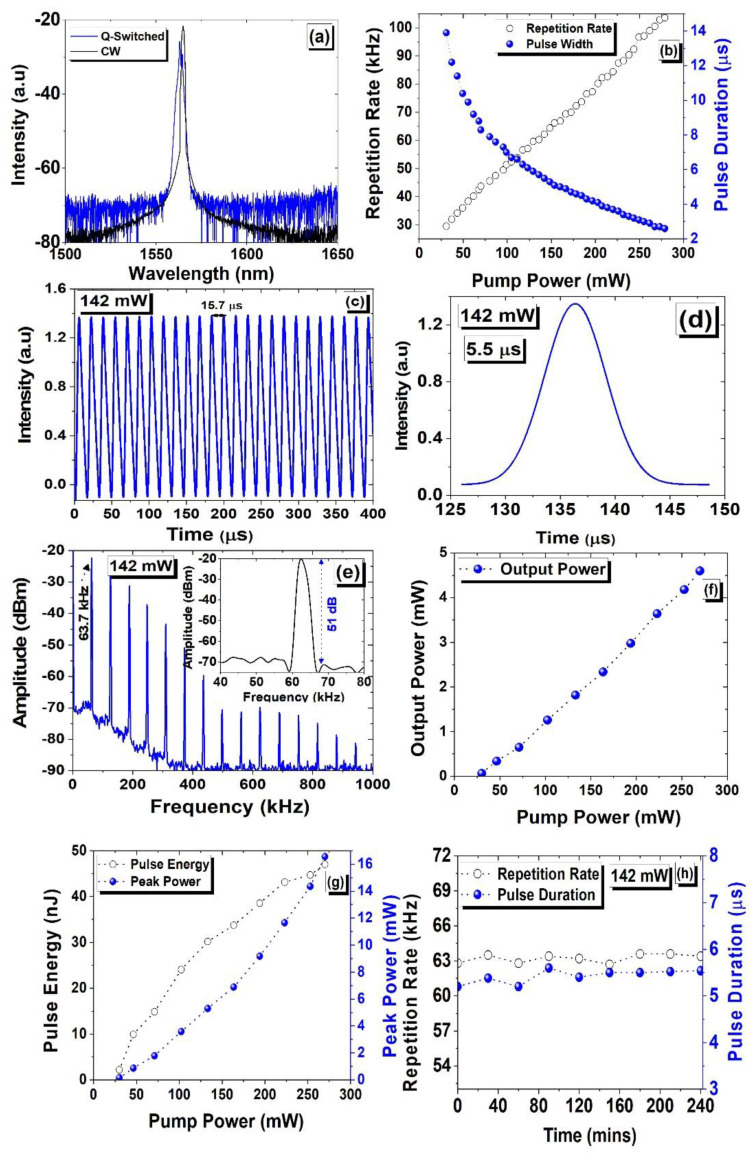
(**a**) A measured emission spectra of EDFL without and with BNC-SA. (**b**) The variation in pulse width (solid blue spheres) and repetition rate (hollow black circles) versus pump. (**c**) The observed optical pulse train under 142 mW of pump power. (**d**) The single pulse spectra under 142 mW of pump power. (**e**) The measured RF spectrum of EDFL with a corresponding pump power of 142 mW with a span of 1000 kHz, an RBW of 3 kHz, and a VBW of 10 Hz; the inset shows a 51 dB SNR. (**f**) The measured output power versus pump power. (**g**) Measured peak power and pulse energy versus pump power. (**h**) The measured pulse duration and repetition rate versus time.

Initially, with an increasing pump up to 30.8 mW (lasing threshold), an EDFL with BNC-SA operates in the CW region. However, with a pump exceeding 30.8 mW, a stable Q-switching is acquired up to 278.96 mW of the pump. Figure 7 illustrates the Q-switching characteristics of the EDFL subject to BNC-SA. Figure 7a depicts the optical spectra at 142 mW of the pump. Before the incorporation of SA, a center wavelength of the EDFL was measured at 1564.84 nm. However, when BNC-SA was fixed inside the laser cavity, the peak wavelength changed to 1562.94 nm. As compared to the optical spectrum produced without SA, there is a significant blue shift in the peak wavelength of 1.9 nm with SA incorporated within the laser cavity, which is attributed to the significant SA loss inside the laser cavity [29,30]. This behavior indicates that modulation characteristics arise as the laser operation jumps to a Q-switching operation from CW mode. Figure 7b presents the measured pulse width and pulse repetition data versus the pump that varied from 30.8 to 278.96 mW. The repetition rate increases gradually from 29.5 to 103.6 kHz with the pump increasing from 30.8 to 278.96 mW, however a pulse width reduces from 13.9 to 2.6 µs. The significant reduction in pulse duration and the increase in pulse repetition are consistent with pump power confirming the Q-switching mechanism. With a maximum pump of 278.96 mW, a minimum pulse duration of 2.6 µs, and a maximum pulse repetition of 103.6 kHz are accomplished. The repetition rate and corresponding pulse width tuning depend strongly on the cavity design; hence, the performance of EDFLs can also be further optimized by changing the overall cavity length. The measured pulse pattern and single pulse under 142 mW of pump power are depicted in Figure 7c and Figure 7d, respectively. These results show that at a particular pump of 142 mW, an adjacent pulse interval of 15.7 µs and a pulse duration of 5.5 µs was achieved. Furthermore, the pulse frequency spectrum of EDFL is shown in Figure 7e. The recorded spectra indicate the fundamental frequency at 63.7 kHz with a maximum SNR of 51 dB (refer to the inset of Figure 7e). The RF spectra showed 15 frequency harmonics, which suggested a steady Q-switching function. Furthermore, a steady pulse train with a 15.7 µs pulse time interval at 142 mW of pump power is presented in Figure 7c, which corresponds well with the repetition rate of 63.7 kHz. To characterize the performance of fiber lasers, the average output power is crucial parameter. Figure 7f–g shows the output power, peak power, and pulse energy data of the EDFL subject to BCN-SA versus pump ranging from 30.8 to 278.96 mW. Based on BNC-SA, the output power shows a linear trend as a function of the pump. A maximum output power of 4.6 mW was attained at the maximum pump of 278.96 mW. The output power was further used to deduce the pulse energy and corresponding peak power of the laser system. The measured results are depicted in Figure 7g, showing a linear trend that agrees with the characteristics of the Q-switching mechanism. At a maximum pump of 278.96 mW, the pulse energy and peak power of the system were noticed to be 47 nJ and 16.56 mW, respectively. The mode-locking mechanism was not observed in the proposed cavity. However, we believe that optimizing the cavity configuration could enable the mode-locking mechanism in EDFLs, subject to BNC-SA. The damage threshold of any SA is another important parameter in the operation of lasers, particularly in pulsed lasers that reflects the quality of ultrashort optical pulses. To investigate the damage threshold of BNC-SA in EDFL, when the pump exceeds 278.96 mW, the pulse operation disappears, and the laser operates in CW mode. However, when the pump power decreases to 278.96 mW, then again, the Q-switched operation starts. This shows that BNC-SA has a damage threshold much higher than 278.96 mW, and this material can be used for the development of ultrastable and high-threshold-based SAs. Stability is another important aspect that impacts the performance of the fiber lasers. Here, the EDFL system’s stability was evaluated by maintaining 80 mW of fixed pump power for a continuous time of 4 h. Figure 7h shows the measured pulse width, and a repetition rate over 4 h, with measurements taken after every 30 min of the time interval. The experimental results indicate that no significant shift in repetition rate, or pulse width was noticed. This shows that the performance of the system was well established throughout the experiment, which confirms that BNC-SA is suitable for stable as well as long-term laser operation in ambient conditions.

Finally, a comparison of EDFL performance based on Al_2_H_2_O_6_Si with other reported SAs materials such as SiOC [31], Al_2_O_3_ [32], Al [33], Si [34], and aluminum zinc oxide [35] is presented in Table 1. As no Q-switching mechanism has been reported based on Al_2_H_2_O_6_Si, various other SAs based on Si, Al, and SiO have been used for a comparative study. The measured results demonstrate that the pulse duration of the EDFL subject to BNC-SA is comparable to previously reported SAs [31,32,33,34,35]. However, the repetition rates (103.6 kHz) and average output power (4.6 mW) are higher than other SAs, which indicates the efficacy of bentonite clay in acting as an SA for the generation of pulse operation. It is also pertinent to mention here that the Q-switching threshold is also lower for EDFLs based on BNC-SA relative to other SAs [31,32,33,34,35].

## 7. Conclusions

To summarize, stable Q-switching in an EDFL was achieved by depositing the powder of a BNC-based SA on a fiber facet by employing a simple deposition method. As the BNC-SA was implemented within the cavity, the Q-switching mechanism was initiated, and by changing the pump from 30.8 mW to 278.96 mW, an increase in the repetition frequency from 29.5 kHz to 103.6 kHz was observed, and the minimum pulse width of 2.6 µs was recorded. The Q-switched EDFL yielded an output power of 4.6 mW with a corresponding peak power of 16.56 mW and pulse energy of 47 nJ at a maximum pump power of 278.96 mW. Furthermore, a highest of 51 dB SNR was acquired, showing a better stability that suggests the BNC material holds significant potential for ultrafast photonics applications.

## Figures and Tables

**Table 1:** Comparative analysis of laser performance subject to various SAs.

SA-Material	Integration Method	Pulse Duration (μs)	Repetition Rates (kHz)	Peak Power (mW)	Average Power (mW)	Pulse Energy (nJ)	Q-Switching Threshold- (mW)	Q-Switching Range (mW)	SNR (dB)	Ref
SiOC	PVA-Film	2.1	96.7	-	2.4	25	111.1	111.1–198	70	[31]
Al_2_O_3_	Nanoparticles	2.8	81	-	-	56.7	158	158–330	56	[32]
Al	Nanoparticles	2.17	48.8	-	0.55	11.29	156	156–300	45	[33]
Si	PVA-Film	2.32	58.7	6.3	0.89	-	41.5	41.5–164	-	[34]
Aluminium zinc oxide	PVA-Film	2.2	86	21.5	4.1	47.3	45.3	45.3–198	69	[35]
Al_2_H_2_O_6_Si	Nanoparticles	2.6	103.6	16.56	4.6	47	30.8	30.8–278.96	51	This work

## Data Availability

Data will be made available on request.
